# Incorporation of Molecular Reorientation into Modeling Surface Pressure-Area Isotherms of Langmuir Monolayers

**DOI:** 10.3390/molecules26144372

**Published:** 2021-07-20

**Authors:** José Agudelo, Guilherme Volpe Bossa, Sylvio May

**Affiliations:** 1 Department of Physics, North Dakota State University, Fargo, ND 58108-6050, USA; jose.agudelo@ndsu.edu; 2 Department of Physics, Institute of Biosciences, Humanities and Exact Sciences, São Paulo State University (UNESP), São José do Rio Preto 15054-000, SP, Brazil; guilherme.vbossa@gmail.com

**Keywords:** Langmuir monolayer, isotherm, lateral pressure, free energy, reorientation, cyclosporin A, nystatin

## Abstract

Langmuir monolayers can be assembled from molecules that change from a low-energy orientation occupying a large cross-sectional area to a high-energy orientation of small cross-sectional area as the lateral pressure grows. Examples include cyclosporin A, amphotericin B, nystatin, certain alpha-helical peptides, cholesterol oxydation products, dumbbell-shaped amphiphiles, organic–inorganic nanoparticles and hybrid molecular films. The transition between the two orientations leads to a shoulder in the surface pressure-area isotherm. We propose a theoretical model that describes the shoulder and can be used to extract the energy cost per molecule for the reorientation. Our two-state model is based on a lattice–sublattice approximation that hosts the two orientations and a corresponding free energy expression which we minimize with respect to the orientational distribution. Inter-molecular interactions other than steric repulsion are ignored. We provide an analysis of the model, including an analytic solution for one specific lateral pressure near a point of inflection in the surface pressure-area isotherm, and an approximate solution for the entire range of the lateral pressures. We also use our model to estimate energy costs associated with orientational transitions from previously reported experimental surface pressure-area isotherms.

## 1. Introduction

Langmuir monolayers at the air–water interface can be formed with a plethora of amphiphiles or surface-active molecules. Changing the lateral pressure and recording the resulting area of the monolayer at given temperature yields isotherms that contain information about molecular interactions, self-assembly, and phase behaviors [1,2,3,4]. The method is established for decades and has contributed significantly to the understanding of lipid layers [5,6,7] (especially the interaction of phospholipids with cholesterol [8]) as well as to the adsorption of protein [9] and DNA [10] onto thin films. It is, generally, not straightforward to deduce molecular properties and interactions from measured surface pressure-area isotherms. To facilitate their interpretation, a considerable body of theoretical studies has accumulated about domain formation [11,12,13] and phase transitions [14,15], biomolecule adsorption onto monolayers [16,17,18], and electrostatic contributions to the lateral pressure [19]. Computer simulations provide additional opportunities to study how isotherms relate to molecular properties of surface-active molecules [20].

The surface pressure-area isotherms of many molecules at the air–water interface are affected (and in some cases likely dominated) by an orientational response, where the molecules are able to reside in distinct states with different cross-sectional areas. Increasing the lateral pressure then leads to a change in the orientational distribution and a characteristic shoulder in the recorded isotherm. A shoulder is a region in the surface pressure-area isotherm where the magnitude of the slope adopts a pronounced and yet non-vanishing minimum. There are many examples for such shoulders and their interpretation in terms of orientational changes. Cyclosporin A [21], amphotericin B [22,23], nystatin [24], stearylspermine [25], monoacylated β-cyclodextrins [26], cholesterol derivatives such as 7α-hydroxycholesterol [27] or oxysterol [28,29], carboxylic acid with a symmetrical triphenylbenzene ring system [30], certain dumbbell-like molecules like amphiphilic bistable rotaxanes [31] or polyoxometalate (POM)-based inorganic–organic–inorganic molecular hybrids [32], tri-podal amphiphiles [33], and alpha-helical peptides [34,35,36] have all been reported to undergo orientational adjustments—typically between two orientations—when being used as surface-active molecules in Langmuir monolayers at varying lateral pressure. Other examples include organic–inorganic nanoparticles such as derivatives of varying amphiphilicity of fully condensed polyhedral oligomeric silsesquioxanes (POSS) [37], matrix organosilane amphiphiles (and also mixed monolayers of vitamin $B_{12}$ mimics with these organosilane amphiphiles) [38], and organic–inorganic hybrid molecular films consisting of a Keggin-type polyoxometalate PW${}_{12}$ and a series of gemini amphiphiles with various lengths of the flexible spacers [39]. While the width of the shoulder allows the two cross-sectional areas of the molecule to be roughly estimated, no attempts have been made to also extract the energy difference associated with the two molecular orientations (we will refer to this energy difference as λ). Indeed, the complexity of the orientation-dependent interactions of surface-active molecules with the air–water interface as well as among the surface-active molecules make the extraction of λ challenging. However, if the lateral pressure profile of the Langmuir monolayer is dominated by the orientational response, with inter-molecular interactions being of secondary importance, λ can be estimated from a simple theoretical model. Presenting this model is the goal of the present work.

In the present work, we propose a theoretical model for the surface pressure-area isotherms of Langmuir monolayers. The monolayer consists of molecules that are able to change from a low-energy orientation that occupies a large cross-sectional area to a high-energy orientation of small cross-sectional area. As the lateral pressure is increased, more and more molecules undergo this reorientation. Our model is based on a lattice-sublattice representation for the air–water interface that hosts the two molecular orientations. We minimize an appropriate free energy expression with respect to the orientational distribution and derive the corresponding surface pressure-area isotherm, including its points of inflection. The isotherm can be calculated analytically for one location that is close to a point of inflection. Because, on the one hand, the lateral pressure at that location can be related directly to the energy difference λ, and, on the other hand, points of inflection can easily be identified from experimentally recorded isotherms, we obtain a versatile and, to the best of our knowledge, previously unrecognized tool to estimate λ (the energy difference between the two molecular orientations) immediately from experimental data. In spite of the highly approximate nature of our model, which exclusively accounts for steric interactions, we expect that the ensuing estimates for λ make a meaningful addition to the information that can be extracted from certain surface pressure-area isotherms.

## 2. Theory

We consider a monolayer composed of amphiphiles (or other surface-active molecules) on the air–water interface, represented by a flat surface of fixed total lateral area *A*. The monolayer contains $N_{0}$ molecules that each can exist in one of two configurations, one lying flat (“horizontal” to the monolayer) and the other standing upright (“vertical” to the monolayer). The former is of lower energy and occupies a larger cross-sectional area. The latter has higher energy and a smaller cross-sectional area. Hence, when the available total area *A* of the monolayer decreases, more molecules are forced to reorient from horizontal to vertical.

**Figure 1:**
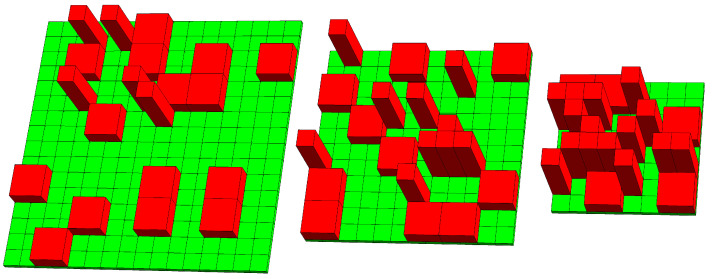
Schematic illustration of an air–water interface, represented by the green-colored lattice that hosts a monolayer of amphiphiles or surface-active molecules (colored red) in two conformations: one that is oriented horizontally and another one vertically to the lattice. As the available area of the monolayer shrinks due to a larger lateral pressure (from the left to the right diagram), more molecules are forced to adopt the vertical orientation, even if the reorientation is associated with an energy cost.

The three images in Figure 1 illustrate the orientational adjustment of the monolayer: most molecules displayed on the left diagram are in their horizontal orientation, and those in the right diagram are in their vertical orientation. The middle diagram shows a monolayer configuration with the same number of molecules being oriented horizontally and vertically. As the available total area *A* decreases, the lateral pressure Π will increase. In order to calculate the isotherm Π(A), which is the main goal of the present work, we will adopt three major approximations: (i) the use of a lattice–sublattice model, (ii) the focus on only two conformations (one oriented horizontally and the other vertically) of each molecule, and (iii) the neglect of inter-molecular interactions beyond the steric repulsion. These three approximations aim to make the model as simple and transparent as possible, thus allowing us to focus on the molecular reorientation without the interference of other physical mechanisms such as electrostatic interactions, domain formation, or lateral phase transitions.

### 2.1. Lattice–Sublattice Model

**Figure 2:**
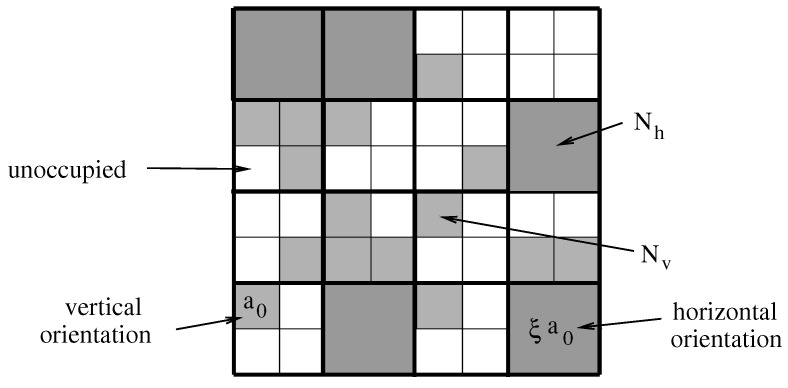
Schematic illustration of the two-dimensional lattice and sublattice that we use to approximate the number of states of the monolayer. The snapshot shows one specific configuration for M=64, ξ=4, $N_{h}=5$, and $N_{v}=15$.

The lattice-sublattice model that we employ in this work was introduced by Han and coworkers [40] and used subsequently by others [41,42,43] to model electrolytes and ionic liquids of asymmetric ion sizes. The model employs a lattice—in our implementation a two-dimensional cubic lattice—consisting of *M* unit cells, each of which has a cross-sectional area $a_{0}=A/M$. A unit cell can host a single amphiphilic molecule in its vertical conformation. The lattice is partitioned into a sublattice consisting of M/ξ unit cells, each of which has a cross-sectional area $\xi a_{0}$. A unit cell of the sublattice can host a single amphiphilic molecule in its horizontal conformation. Note that ξ is the ratio between the unit cell’s cross-sectional areas of the sublattice and the actual lattice. It thus reflects the ratio between the cross-sectional molecular areas of the amphiphile in the horizontal and vertical orientations. Assume that of the

(1) $$N_{0}=N_{h}+N_{v}$$

amphiphilic molecules that reside on the monolayer, $N_{h}$ and $N_{v}$ molecules are in their horizontal (index “*h*”) and vertical (index “*v*”) orientation, respectively. The molecules are always arranged so as to avoid steric overlap. That is, exactly $\xi N_{h}+N_{v}$ lattice sites of the *M* total sites are occupied by amphiphilic molecules, and the remaining $M-\xi N_{h}-N_{v}$ sites remain unoccupied. The corresponding fraction of occupied versus total lattice sites is

(2) $$\phi_{tot}=\frac{\xi N_{h}+N_{v}}{M}=\phi_{h}+\phi_{v},$$

which we express as the sum of the two individual contributions

(3) $$\begin{array}{c} \phi_{h}=\xi \frac{N_{h}}{M},\;\phi_{v}=\frac{N_{v}}{M}, \end{array}$$

originating from molecules in their horizontal and vertical orientation, respectively. While $\phi_{tot}$ will change as function of the orientational distribution, the scaled number of molecules

(4) $$\begin{array}{c} \phi_{0}=\frac{N_{0}}{M}=\frac{\phi_{h}}{\xi }+\phi_{v} \end{array}$$

is a fixed constant, given that no molecules are added or removed from the monolayer. Note that $\phi_{0}$ can also be interpreted as the fraction of lattice sites occupied by the molecules, given they would all reside in their vertical orientation; hence, $\phi_{0}\leq \phi_{tot}$. The lattice model is illustrated in Figure 2 for the example ξ=4.

Note that the specific configuration displayed in Figure 2 (which has M=64, $N_{v}=5$, $N_{h}=15$, $\phi_{h}=20/64$, $\phi_{v}=15/64$, $\phi_{tot}=35/64$, and $\phi_{0}=20/64$) corresponds to that of the right diagram in Figure 1.

### 2.2. Free Energy and Lateral Pressure

At fixed temperature *T* and total area *A*, the monolayer will adjust its orientational distribution so as to minimize the Helmholtz free energy F=U−TS. Recall that we assume molecules in the vertical orientation to possess a different energy than those in the horizontal orientation. In addition, we also assume the absence of inter-molecular interactions. Hence, we can express the internal energy of the monolayer as

(5) $$U=\lambda N_{v},$$

where λ characterizes the energy cost to switch a single molecule from its horizontal to its vertical orientation.

The number of distinguishable states that the $N_{0}$ amphiphiles are able to adopt on the lattice is given by the number of ways to arrange $N_{h}$ molecules on the available M/ξ sublattice sites times the number of ways to arrange $N_{v}$ molecules on the remaining $M-\xi N_{h}$ lattice sites [40],

(6) $$\Omega =\frac{\left(\frac{M}{\xi }\right)!}{\left(\frac{M}{\xi }-N_{h}\right)!\;N_{h}!}\times \frac{(M-\xi N_{h})!}{\left(M-\xi N_{h}-N_{v}\right)!\;N_{v}!}.$$

Using Stirling’s approximation ln(x!)≈xlnx−x, which is valid for x≫1, we express the entropy $S=k_{B}\ln\Omega$ of the amphiphilic molecules (where $k_{B}$ is Boltzmann’s constant) as

(7) $$\begin{array}{ccc} -\frac{S}{k_{B}M} & = & \frac{\phi_{h}}{\xi }\ln\phi_{h}+\left(\frac{1}{\xi }-1\right)\left(1-\phi_{h}\right)\ln\left(1-\phi_{h}\right)\\ & + & \left(1-\phi_{h}-\phi_{v}\right)\ln\left(1-\phi_{h}-\phi_{v}\right)+\phi_{v}\ln\phi_{v}. \end{array}$$

Inserting *U* and *S* into the free energy *F* leads to our final expression

(8) $$\begin{array}{ccc} f(\phi_{h},\phi_{v}) & = & \lambda \phi_{v}+\frac{\phi_{h}}{\xi }\ln\phi_{h}+\left(\frac{1}{\xi }-1\right)\left(1-\phi_{h}\right)\ln\left(1-\phi_{h}\right)\\ & + & \left(1-\phi_{h}-\phi_{v}\right)\ln\left(1-\phi_{h}-\phi_{v}\right)+\phi_{v}\ln\phi_{v} \end{array}$$

for the free energy per lattice site, f=F/M, expressed as function of the two fractions of occupied lattice sites $\phi_{h}$ and $\phi_{v}$. Note that here and in the following we use $k_{B}T$ as our unit of energy. Note also that we have included only translational entropy contributions into *S*. Hence, λ may (and usually will) include entropic contributions that originate from changes in orientational fluctuations, internal conformational degrees of freedom, and hydrophobic interactions of the molecule when changing its orientation. Of course, when taking the derivative $-{\left(\partial F/\partial T\right)}_{A,N_{0}}$, we will only recover the translational entropy. We do not make any statement about the temperature dependence of λ in this work.

Next, we use the function $f(\phi_{h},\phi_{v})$ to calculate the lateral pressure of the monolayer. In analogy to the ordinary pressure, we define the lateral pressure Π that acts within the monolayer by

(9) $$\Pi =-{\left(\frac{\partial F}{\partial A}\right)}_{T,N_{0}}.$$

That is, we take the derivative of *F* with respect to the total lateral area *A* of the monolayer at fixed temperature *T* and at fixed number of molecules $N_{0}$. The molecules will adjust their orientational distribution so as to minimize *F*, implying that the function $f(\phi_{h},\phi_{v})$ in Equation (8) will be minimal with respect to $\phi_{h}$ and $\phi_{v}$, subject to the conservation of molecules as dictated by $\phi_{0}=\phi_{h}/\xi +\phi_{v}$; see Equation (4). We can express this optimized function as $f^{opt}\left(\phi_{0}\right)$. Using $F=Af^{opt}\left(\phi_{0}\right)/a_{0}$ and $\phi_{0}=N_{0}a_{0}/A$ allows us to re-express the scaled lateral pressure as

(10) $$a_{0}\Pi =\phi_{0}\frac{df^{opt}\left(\phi_{0}\right)}{d\phi_{0}}-f^{opt}\left(\phi_{0}\right).$$

If the function $f^{opt}\left(\phi_{0}\right)$ is known, we can calculate the lateral pressure immediately from Equation (10) and plot it as function of the scaled area per molecule $a/a_{0}=1/\phi_{0}$, where $a=A/N_{0}$ is the average area a molecule occupies on the monolayer. In the limit $a/a_{0}\to 1$, we approach closest packing where all molecules must be in the vertical orientation. We obtain the function $f^{opt}\left(\phi_{0}\right)$ from $f(\phi_{h},\phi_{v})$ by inserting the relation $\phi_{h}=\xi \;\left(\phi_{0}-\phi_{v}\right)$ (see Equation (4)) and subsequently solving the equation

(11) $$\frac{\partial f(\xi \;\left(\phi_{0}-\phi_{v}\right),\phi_{v})}{\partial \phi_{v}}=0$$

with respect to $\phi_{v}$. This results in the equilibrium distribution $\phi_{v}=\phi_{v}^{opt}\left(\phi_{0}\right)$, which we insert back into $f(\xi \;\left(\phi_{0}-\phi_{v}\right),\phi_{v})$. Hence

(12) $$f^{opt}\left(\phi_{0}\right)=f\left(\xi \;\left(\phi_{0}-\phi_{v}^{opt}\left(\phi_{0}\right)\right),\phi_{v}^{opt}\left(\phi_{0}\right)\right).$$

With this, we have specified the calculation of the lateral pressure solely from the function $f(\phi_{h},\phi_{v})$.

### 2.3. The Limiting Case ξ=1

The coupling between the orientational distribution of the molecules and the lateral pressure arises because the cross-sectional areas $a_{0}\xi$ and $a_{0}$ of the horizontal and vertical orientations, respectively, differ as long as ξ≠1. For the special case ξ=1, orientational adjustments decouple from the lateral pressure. We can quantify this case explicitly. According to Equation (4), ξ=1 implies $\phi_{h}=\phi_{0}-\phi_{v}$ and thus $\phi_{tot}=\phi_{0}$. With this, Equation (8) reads

(13) $$f\left(\phi_{0}-\phi_{v},\phi_{v}\right)=\left(\phi_{0}-\phi_{v}\right)\ln\left(\phi_{0}-\phi_{v}\right)+\phi_{v}\ln\left(\phi_{v}\right)+\left(1-\phi_{0}\right)\ln\left(1-\phi_{0}\right)+\lambda \phi_{v}.$$

Minimization of *f* demands solving the equation

(14) $$\frac{\partial f}{\partial \phi_{v}}=\ln\left(\frac{\phi_{v}}{\phi_{0}-\phi_{v}}\right)+\lambda =0,$$

which leaves us with

(15) $$\phi_{v}=\phi_{v}^{opt}=\frac{\phi_{0}}{1+e^{\lambda }}.$$

By inserting this result into Equation (13), we obtain the optimal free energy

(16) $$f^{opt}\left(\phi_{0}\right)=\phi_{0}\ln\phi_{0}+\left(1-\phi_{0}\right)\ln\left(1-\phi_{0}\right)-\phi_{0}\ln\left(1+e^{\lambda }\right).$$

Using Equation (10) we immediately arrive at the (scaled) lateral pressure

(17) $$\begin{array}{c} a_{0}\Pi =-\ln\left(1-\phi_{0}\right). \end{array}$$

This result represents the well-known pressure [44] of a simple lattice gas in the absence of interactions. As expected, it is independent of λ. Changing λ leads to an orientational re-distribution of the molecules. However, this does not change their cross-sectional area, and therefore the lateral pressure is not affected by λ.

## 3. Results and Discussion

In this section, we present and analyze predictions of our model. We first discuss how we obtain numerical results for the optimal orientational distribution $\phi_{v}=\phi_{v}^{opt}\left(\phi_{0}\right)$ and the scaled lateral pressure $a_{0}\Pi$ as a function of $a/a_{0}$. We also specify how to detect points of inflection in the surface pressure-area isotherm. Then, we calculate one point of the isotherm analytically—a point that is not identical but usually resides close to a point of inflection. Based on this, we finally discuss applications of our model to experimentally reported surface pressure-area isotherms, resulting in estimates of ξ and λ for previously investigated surface-active molecules at the air–water interface. We demonstrate that the application is remarkably simple, yielding information in addition to what is usually extracted from isotherms that are dominated by molecular reorientations.

### 3.1. Numerical Results

Recall that the scaled pressure $a_{0}\Pi$ can be calculated from the free energy $f^{opt}\left(\phi_{0}\right)=f\left(\xi \left(\phi_{0}-\phi_{v}^{opt}\left(\phi_{0}\right)\right),\phi_{v}^{opt}\left(\phi_{0}\right)\right)$ if the function $\phi_{v}=\phi_{v}^{opt}\left(\phi_{0}\right)$ is known. If $\phi_{v}^{opt}\left(\phi_{0}\right)$ is not known analytically, but only through a numerical representation (that is, a list of values ${\left\{\phi_{0},\phi_{v}\right\}}_{i}$), it will be inconvenient to carry out derivatives with respect to $\phi_{0}$. We show in the following how to calculate the lateral pressure $a_{0}\Pi$ using only the function $f\left(\phi_{h},\phi_{v}\right)=f\left(\xi \left(\phi_{0}-\phi_{v}\right),\phi_{v}\right)$ and a numerical representation of $\phi_{v}=\phi_{v}^{opt}\left(\phi_{0}\right)$, yet no derivative of that function. To this end, it is convenient to define $\tilde{f}\left(\phi_{0},\phi_{v}\right)=f\left(\xi \left(\phi_{0}-\phi_{v}\right),\phi_{v}\right)$. This function is known explicitly (see Equation (8)), and we can carry out any partial derivatives analytically. Minimizing $\tilde{f}\left(\phi_{0},\phi_{v}\right)$ with respect to $\phi_{v}$, i.e., setting $\partial \tilde{f}\left(\phi_{0},\phi_{v}\right)/\partial \phi_{v}=0$, yields the equation

(18) $$\lambda =\ln\frac{\xi (\phi_{0}-\phi_{v})}{\phi_{v}}+\left(1-\xi \right)\ln\frac{1-\xi (\phi_{0}-\phi_{v})}{1-\phi_{v}-\xi \left(\phi_{0}-\phi_{v}\right)},$$

which defines the function $\phi_{v}=\phi_{v}^{opt}\left(\phi_{0}\right)$ at fixed ξ and λ. Based on Equation (18), this function can be obtained numerically but not analytically (with the exception of the point $\phi_{0}=1/\xi$ as discussed below). Using the chain rule leads to

(19) $$\frac{d\tilde{f}\left(\phi_{0},\phi_{v}\left(\phi_{0}\right)\right)}{d\phi_{0}}=\frac{\partial \tilde{f}\left(\phi_{0},\phi_{v}\right)}{\partial \phi_{0}}+\frac{\partial \tilde{f}\left(\phi_{0},\phi_{v}\right)}{\partial \phi_{v}}\frac{d\phi_{v}}{d\phi_{0}}.$$

Upon taking the full differential of the vanishing function $\partial \tilde{f}\left(\phi_{0},\phi_{v}\right)/\partial \phi_{v}=0$, it follows that

(20) $$\frac{\partial^{2}\tilde{f}\left(\phi_{0},\phi_{v}\right)}{\partial \phi_{v}\partial \phi_{0}}d\phi_{0}+\frac{\partial^{2}\tilde{f}\left(\phi_{0},\phi_{v}\right)}{\partial^{2}\phi_{v}}d\phi_{v}=0,$$

and we obtain an expression for the derivative

(21) $$\frac{d\phi_{v}}{d\phi_{0}}=-\frac{\frac{\partial^{2}\tilde{f}\left(\phi_{0},\phi_{v}\right)}{\partial \phi_{v}\partial \phi_{0}}}{\frac{\partial^{2}\tilde{f}\left(\phi_{0},\phi_{v}\right)}{\partial^{2}\phi_{v}}}$$

in terms of the explicitly known function $\tilde{f}\left(\phi_{0},\phi_{v}\right)$ and the not explicitly known function $\phi_{v}=\phi_{v}^{opt}\left(\phi_{0}\right)$. Thus, combining Equations (10), (19), and (21), we arrive at an expression for the scaled lateral pressure

(22) $$a_{0}\Pi =\phi_{0}\left[\frac{\partial \tilde{f}\left(\phi_{0},\phi_{v}\right)}{\partial \phi_{0}}-\frac{\partial \tilde{f}\left(\phi_{0},\phi_{v}\right)}{\partial \phi_{v}}\frac{\frac{\partial^{2}\tilde{f}\left(\phi_{0},\phi_{v}\right)}{\partial \phi_{v}\partial \phi_{0}}}{\frac{\partial^{2}\tilde{f}\left(\phi_{0},\phi_{v}\right)}{\partial^{2}\phi_{v}}}\right]-\tilde{f}\left(\phi_{0},\phi_{v}\right),$$

valid for any fixed ξ and λ. It is important to realize that all derivatives in Equation (22) are known explicitly. After calculating them, we merely need to insert our numerical representation of the function $\phi_{v}=\phi_{v}^{opt}\left(\phi_{0}\right)$ (that is, the list ${\left\{\phi_{0},\phi_{v}\right\}}_{i}$), and no derivatives need to be taken thereafter.

**Figure 3:**
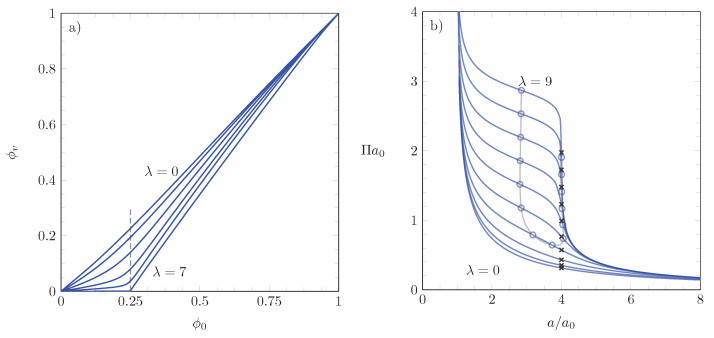
Diagram (**a**): Fraction of lattice sites occupied by molecules in the vertical orientation $\phi_{v}=\phi_{v}^{opt}\left(\phi_{0}\right)$ as a function of the (scaled) number of molecules $\phi_{0}$ for λ=0,1,2,3,4,7 (from top to bottom). Diagram (**b**): Dimensionalized pressure $a_{0}\Pi$ as a function of the scaled area per molecule $a/a_{0}$ for λ=0,1,2,3,4,5,6,7,8,9 (from bottom to top). All results correspond to numerical solutions of Equation (18) and subsequent use of Equation (22). The blue circles mark the positions of the inflection points for each isotherm; the gray line that connects the blue circles marks the set of all inflection points that exist for λ≤9. The black crosses display the lateral pressures at positions $\phi_{0}=1/\xi =a_{0}/a$. All results are derived for ξ=4.

Figure 3 displays our numerical results for ξ=4 and various choices of λ. The left diagram shows the function $\phi_{v}=\phi_{v}^{opt}\left(\phi_{0}\right)$ for λ=0,1,2,3,4,7 (from top to bottom). The right diagram presents the (scaled) lateral pressure $a_{0}\Pi$ for λ=0,1,2,3,4,5,6,7,8,9 (from bottom to top). The surface pressure-area isotherms in the right diagram exhibit shoulders for sufficiently large λ, similar to those reported in experimental work as discussed in the introduction.

### 3.2. Points of Inflection

As we will discuss below, the usefulness of our theoretical model relies on the identification of points of inflection close to the location $a/a_{0}=\xi$. These points are marked by blue circles on the isotherms in the right diagram of Figure 3. The gray line that connects these points marks the location for the points of inflection corresponding to other choices of λ. Given our choice ξ=4, points of inflection exist for λ≥3.85. When the points of inflection are well pronounced and identifiable by visual inspection, the larger of the two for any given isotherm is located close to $a/a_{0}=\xi$. The lateral pressure at position $a/a_{0}=1/\phi_{0}=\xi$ is marked by black crosses on the right diagram of Figure 3.

We discuss briefly how we have identified the points of inflection in Figure 3. As for the calculation of the lateral pressure in Equation (22), we have ensured high numerical accuracy by only using the function $\tilde{f}\left(\phi_{0},\phi_{v}\right)$ (as specified in Equation (8) with $\phi_{h}=\xi \;\left(\phi_{0}-\phi_{v}\right)$) and a numerical representation ${\left\{\phi_{0},\phi_{v}\right\}}_{i}$ of the function $\phi_{v}=\phi_{v}^{opt}\left(\phi_{0}\right)$, yet no derivative of that function. Recall that the key was to express the derivative

(23) $$\frac{df^{opt}\left(\phi_{0}\right)}{d\phi_{0}}=\frac{\partial \tilde{f}\left(\phi_{0},\phi_{v}\right)}{\partial \phi_{0}}-\frac{\partial \tilde{f}\left(\phi_{0},\phi_{v}\right)}{\partial \phi_{v}}\frac{\frac{\partial^{2}\tilde{f}\left(\phi_{0},\phi_{v}\right)}{\partial \phi_{v}\partial \phi_{0}}}{\frac{\partial^{2}\tilde{f}\left(\phi_{0},\phi_{v}\right)}{\partial^{2}\phi_{v}}}$$

as a function of partial derivatives of $\tilde{f}\left(\phi_{0},\phi_{v}\right)$ only and, at the end, to insert $\phi_{v}=\phi_{v}^{opt}\left(\phi_{0}\right)$. The same method can be worked out to find the points of inflection for the lateral pressure $a_{0}\Pi$ when plotted as function of $a/a_{0}=1/\phi_{0}$. If plotted as function of $\phi_{0}$, the points of inflection of the isotherm would be defined by the equation

(24) $$\frac{d^{2}\left(a_{0}\Pi \left(\phi_{0}\right)\right)}{d\phi_{0}^{2}}=\frac{d^{2}f^{opt}\left(\phi_{0}\right)}{d\phi_{0}^{2}}+\phi_{0}\frac{d^{3}f^{opt}\left(\phi_{0}\right)}{d\phi_{0}^{3}}=0.$$

Equation (24) follows immediately from Equation (10). However, if the lateral pressure $\tilde{\Pi }\left(a/a_{0}\right)=\Pi \left(\phi_{0}\right)$ is plotted as function of $a/a_{0}=1/\phi_{0}$, the condition for finding the points of inflection becomes

(25) $$\frac{1}{\phi_{0}^{4}}\;\frac{d^{2}\left(a_{0}\tilde{\Pi }\left(\frac{a}{a_{0}}\right)\right)}{d{\left(\frac{a}{a_{0}}\right)}^{2}}=\frac{d^{2}\left(a_{0}\Pi \left(\phi_{0}\right)\right)}{d\phi_{0}^{2}}+\frac{2}{\phi_{0}}\;\frac{d(a_{0}\Pi \left(\phi_{0}\right))}{d\phi_{0}}=3\frac{d^{2}f^{opt}\left(\phi_{0}\right)}{d\phi_{0}^{2}}+\phi_{0}\frac{d^{3}f^{opt}\left(\phi_{0}\right)}{d\phi_{0}^{3}}=0.$$

To apply this condition, we need to calculate the two derivatives $d^{2}f^{opt}\left(\phi_{0}\right)/d\phi_{0}^{2}$ and $d^{3}f^{opt}\left(\phi_{0}\right)/d\phi_{0}^{3}$. Regarding the former, we take another derivative of Equation (23) and use Equation (21) to eliminate $d\phi_{v}/d\phi_{0}$. The same method can then be applied again to calculate the third derivative from the second.

### 3.3. Analytic Solution for $\phi_{0}=1/\xi$

At the location $\phi_{0}=1/\xi$, we can solve Equation (18) analytically. The solution $\phi_{v}^{☆}=\phi_{v}\left(\phi_{0}=1/\xi \right)$ is given by

(26) $$\phi_{v}^{☆}=\frac{1}{e^{\lambda }\;{\left(\frac{\xi -1}{\xi }\right)}^{\xi -1}+\xi }.$$

The limit ξ→1 indeed recovers $\phi_{v}^{☆}=1/\left(1+e^{\lambda }\right)$ as expected (see Equation (15)). Recall that the lateral pressure can be calculated based on Equation (22), which requires only the insertion of the function $\phi_{v}=\phi_{v}^{opt}\left(\phi_{0}\right)$ but not its derivative. Hence, knowledge of the function $\phi_{v}=\phi_{v}^{opt}\left(\phi_{0}\right)$ at a single point $\phi_{0}$ is sufficient to calculate the lateral pressure at that point. Inserting the point $\phi_{0}=1/\xi$ and $\phi_{v}^{☆}$ according to Equation (26) into Equation (22) yields for the (scaled) lateral pressure

(27) $$a_{0}\Pi =-\ln\left(\xi -1\right)+\frac{1}{\xi }\;\ln\left[e^{\lambda }{\left(\xi -1\right)}^{\xi -1}+\xi^{\xi }\right].$$

This pressure is exactly what our model predicts at $\phi_{0}=1/\xi$. In Figure 3, we have marked the lateral pressures $a_{0}\Pi$ at positions $\phi_{0}=1/\xi$ by black crosses.

**Figure 4:**
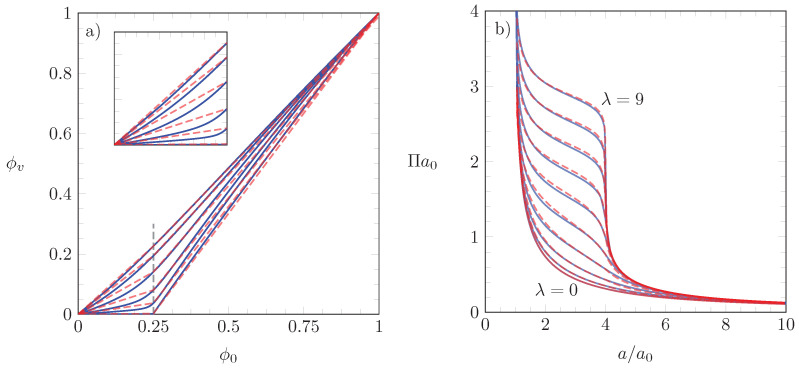
The blue lines in both diagrams re-plot the blue lines already shown in Figure 3. In addition, the dashed red lines show the piece-wise linear approximation for $\phi_{v}\left(\phi_{0}\right)$ in Equation (28) (diagram **a**) and the corresponding scaled lateral pressure $a_{0}\Pi$ (diagram **b**). The inset in diagram **a** is a magnified view of the region $0\leq \phi_{0}\leq 0.25$. As in Figure 3, all results are derived for ξ=4.

In order to produce a reasonable approximation also for $\phi_{0}\neq \left(1/\xi \right)$, we may simply adopt linear behaviors for the function

(28) $$\phi_{v}=\left\{\begin{array}{cc} \xi \phi_{v}^{*}\phi_{0} & \mathrm{if}\;0\leq \phi_{0}\leq \frac{1}{\xi },\\ \phi_{v}^{*}+\frac{1-\phi_{v}^{*}}{1-\frac{1}{\xi }}\left(\phi_{0}-\frac{1}{\xi }\right) & \mathrm{if}\;\frac{1}{\xi }\leq \phi_{0}\leq 1, \end{array}\right.$$

in the two regions $0\leq \phi_{0}<1/\xi$ and $1/\xi <\phi_{0}\leq 1$. The red dashed lines in the left diagram of Figure 4 show the piece-wise linear approximations for the function $\phi_{v}\left(\phi_{0}\right)$ according to Equation (28).

The corresponding lateral pressure obtained by inserting $\phi_{v}\left(\phi_{0}\right)$ according to Equation (28) into Equation (22) is shown in the right diagram of Figure 4 by the red dashed lines. The blue lines in Figure 4 reproduce the numerical solutions already shown in Figure 3. The matching between the two sets of isotherms is reasonable, suggesting that Equation (28) is a meaningful approximation.

### 3.4. Application of our Theoretical Model to Experimental Isotherms

**Figure 5:**
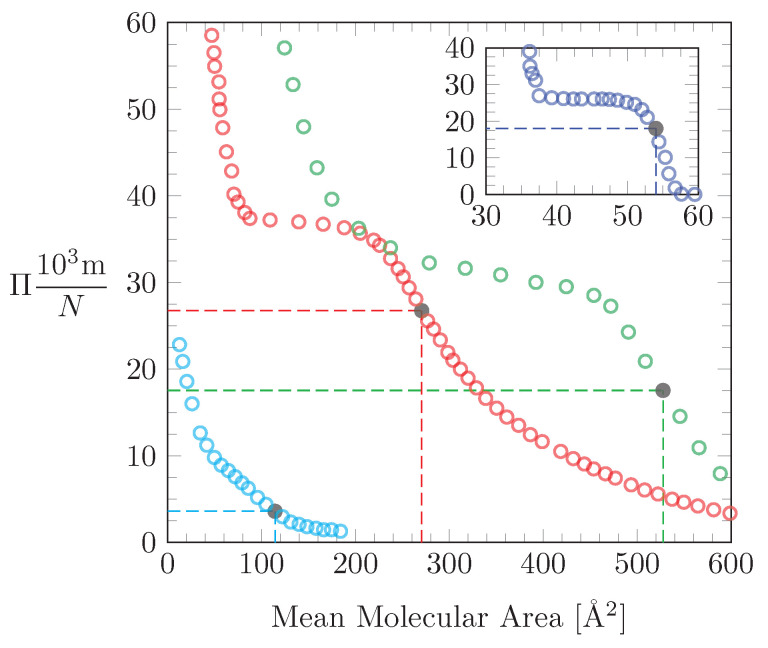
Four surface pressure-area isotherms are reproduced from previously reported studies: The red-colored data set is for cyclosporin A in the presence of 3 M NaCl, adopted from Figure 5 of Miñones et al. [21]. The lightblue-colored data set is for a monolayer of the antifungal drug nystatin, adopted from Figure 1 of Hąc-Wydro and Dynarowicz-ątka [24]. The green-colored data set is for a monolayer composed of the synthetic alpha-helical lipopeptide BBC16, adopted from Figure 1 of Strzalka et al. [35]. The blue-colored data set shown in the inset is for 7α-hydroxycholesterol, adopted from Figure 2A of Wnętrzak et al. [27]. For all four isotherms, we have marked the relevant point of inflection with a filled black circle.

Here, we demonstrate how to use our theoretical model to estimate ξ and λ from experimentally reported isotherms. The isotherm under consideration should correspond to a monolayer of surface-active molecules that undergo a molecular reorientation between two dominating orientational states as the lateral pressure changes. Application of our theoretical model requires us to identify a point of inflection in the isotherms (that is, the first point of inflection as the mean molecular area is decreased). Figure 5 shows four examples of isotherms where our model may find application.

The first example is represented by the red circles in Figure 5 and is adopted from Miñones et al. [21], who investigated monolayers of the cyclic oligopeptide cyclosporin A at the air–water interface. The isotherm was recorded at high ionic strength (3 M NaCl) and was interpreted by the authors in terms of two orientations that cyclosporin A adopts, one in which the ring of the molecule lies horizontally on the surface and another where the ring is shifted to a vertical orientation. A collapse area $a_{0}\approx 0.4\;{\mathrm{nm}}^{2}$, a point of inflection at a mean molecular area $a=2.7\;{\mathrm{nm}}^{2}$, and a corresponding lateral pressure Π=27 mN/m can be identified by visual inspection. The point of inflection is highlighted in Figure 5 by a filled black circle. We obtain $\xi =a/a_{0}=6.75$ and $a_{0}\Pi =2.61$. (Recall that because of the scaling by the thermal energy unit this actually means $a_{0}\Pi /k_{B}T=0.4\;{\mathrm{nm}}^{2}\;27\;\mathrm{mN}/\text{m}/\left(4.14\times 10^{-21}\;J\right)=2.61$.) We now solve Equation (27) for λ, yielding

(29) $$\lambda =\ln\frac{{\left(\xi -1\right)}^{\xi }\;e^{\xi a_{0}\Pi }-\xi^{\xi }}{{\left(\xi -1\right)}^{\xi -1}},$$

which will have a real-valued solution given that $\xi >1/(1-e^{-a_{0}\Pi })$. Inserting ξ and $a_{0}\Pi$ into Equation (29) results in λ=19. (Recall that this is in units of $k_{B}T$; $19\;k_{B}T$ correspond to 7.8 kJ/mol.) Hence, given the experimentally reported surface pressure-area isotherm reflects a molecular reorientation of cyclosporin A, our model estimates an energy cost of about $19\;k_{B}T$ for that reorientation.

The second example applies to the antifungal drug nystatin, a polyene macrolide derived from the bacterium *streptomyces noursei*. Hąc-Wydro and Dynarowicz-ątka [24] have recorded isotherms of Langmuir monolayers composed of nystatin and suggested that the molecule changes from horizontal to vertical orientation with increasing lateral pressure. The surface pressure-area isotherm from Figure 1 of the original publication [24] is reproduced by the lightblue circles in Figure 5. We estimate $a_{0}=0.1\;{\mathrm{nm}}^{2}$ as well as $a=1.15\;{\mathrm{nm}}^{2}$ and Π=4 mN/m at the inflection point. This yields ξ=11.5 and $a_{0}\Pi =0.097$, implying λ=0.7. The predicted value of λ is small, which is also suggested by the weakly developed shoulder in the isotherm.

The third example, represented by the green circles in Figure 5, reproduces a surface pressure-area isotherm of a monolayer composed of the synthetic alpha-helical lipopeptide BBC16. The displayed isotherm was recorded by Strzalka et al. [35] (see Figure 1 in that publication) and rationalized in terms of a pressure-induced reorientation of the peptide. At low pressures the peptide is oriented with its long axis parallel to the interface, whereas at high pressures the peptide changes its orientation to be normal to the interface. We extract $a_{0}=1.0\;{\mathrm{nm}}^{2}$ and a point of inflection at $a=5.25\;{\mathrm{nm}}^{2}$ with a pressure of Π=18 mN/m. This gives rise to $\xi =a/a_{0}=5.25$, $a_{0}\Pi =4.35$ and thus λ=24.

Our final example applies to the molecule 7α-hydroxycholesterol, a cholesterol oxidation product, which at low pressure forms a monolayer that is anchored to the air–water interface with its two OH groups. Upon increasing the lateral pressure the sterol switches from horizontal to vertical orientation, with one OH group detaching from the air–water interface. The blue-colored data in the inset of Figure 5 reproduce an isotherm that was recorded by Wnętrzak et al. [27] (Figure 2A in that publication). We extract $a_{0}=0.36\;{\mathrm{nm}}^{2}$ and a point of inflection at $a=0.54\;{\mathrm{nm}}^{2}$ with a pressure of Π=18 mN/m. This leads to $\xi =a/a_{0}=1.5$, $a_{0}\Pi =1.57$, implying λ=1.0.

The ability to estimate λ is the main accomplishment of our model. We highlight its simplicity: the three values $a_{0}$, *a*, and Π can be extracted from a given isotherm, and this leads immediately $\xi =a/a_{0}$ and to λ through Equation (29). We also point out that our model involves a number of assumptions that will not be exactly fulfilled in a real system. First, we keep the number of molecules $N_{0}$ strictly constant. In a real system, molecules can be pushed out of the monolayer, which can lead to hysteresis effects [21]. Second, many monolayer isotherms are known to reflect macroscopic phase separation or the formation of domains. Domain formation is usually induced by attractive interactions between the amphiphiles. Our theoretical model does not account for attractive interactions. The present model may, in principle, be extended to include attractive interactions on the mean-field level (this would generalize the Bragg-Williams model [44]). However, such an extension may no longer allow for an interpretation as simple as in the present case. Third, we have adopted simple geometric approximations for the amphiphilic molecule and a lattice–sublattice approximation to calculate the entropy in Equation (7). These uncertainties and approximations call for caution when specifying the magnitude of λ. Nevertheless, our model does extract an additional piece of information (namely the energy cost λ to change the orientation of the surface-active molecule under consideration) that has not been made use of in previous work.

## 4. Conclusions and Outlook

The theoretical model for the surface pressure-area isotherm of Langmuir monolayers that we present in this work targets surface-active molecules that undergo an orientational change in response to a sufficiently large lateral pressure. The model makes an explicit prediction for a point on the isotherm that is close to the first point of inflection as the lateral pressure increases. Knowing the ratio of the cross-sectional areas of the two molecular orientations involved ($\xi =a/a_{0}$) and the lateral pressure at the point of inflection yields a prediction for the energy difference λ between these two states; see Equation (29). The estimated value of λ must be regarded as an approximation because our model is based on a number of assumptions such as the presence of exactly two dominating molecular orientations, a lattice–sublattice representation of the air–water interface, and the negligibility of inter-molecular interactions other than steric ones. Inter-molecular interactions (most notably dipole–dipole interactions [45]), often lead to phase transitions in monolayers (for example, into a liquid-expanded and a liquid-condensed phase [1]). We emphasize that the description of phase transitions is not within the scope of the present study. Including inter-molecular interactions on a mean-field level may lead to a useful extension of the present model. Nevertheless, in spite of its approximate nature, our present model adds a simple method to analyze certain isotherms and extract an estimate of a molecular property that, to the best of our knowledge, has remained unrecognized in the past. 

## Data Availability

Not applicable.
